# Artificial Intelligence and Circulating Cell-Free DNA Methylation Profiling: Mechanism and Detection of Alzheimer’s Disease

**DOI:** 10.3390/cells11111744

**Published:** 2022-05-25

**Authors:** Ray O. Bahado-Singh, Uppala Radhakrishna, Juozas Gordevičius, Buket Aydas, Ali Yilmaz, Faryal Jafar, Khaled Imam, Michael Maddens, Kshetra Challapalli, Raghu P. Metpally, Wade H. Berrettini, Richard C. Crist, Stewart F. Graham, Sangeetha Vishweswaraiah

**Affiliations:** 1 Department of Obstetrics and Gynecology, Oakland University-William Beaumont School of Medicine, Royal Oak, MI 48309, USA; ray.bahado-singh@beaumont.org (R.O.B.-S.); ali.yilmaz@beaumont.org (A.Y.); stewart.graham@beaumont.edu (S.F.G.); 2 Department of Obstetrics and Gynecology, Beaumont Health, 3601 W. 13 Mile Road, Royal Oak, MI 48073, USA; dr.faryaljafar@gmail.com (F.J.); drkshetra93@gmail.com (K.C.); 3 Vugene, LLC, 625 Kenmoor Ave Suite 301 PMB 96578, Grand Rapids, MI 49546, USA; juozas@vugene.us; 4 Department of Care Management Analytics, Blue Cross Blue Shield of Michigan, Detroit, MI 48226, USA; buketaydas@gmail.com; 5 Department of Alzheimer’s Disease Research, Beaumont Research Institute, 3811 W. 13 Mile Road, Royal Oak, MI 48073, USA; 6 Department of Internal Medicine, Beaumont Health, 3601 W. 13 Mile Road, Royal Oak, MI 48073, USA; khaled.imam@beaumont.edu (K.I.); mmaddens@beaumont.edu (M.M.); 7 Department of Molecular and Functional Genomics, Geisinger, Danville, PA 17821, USA; mrprao@gmail.com (R.P.M.); wadeb@pennmedicine.upenn.edu (W.H.B.); 8 Department of Psychiatry, Perelman School of Medicine, University of Pennsylvania, Philadelphia, PA 19104, USA; crist@pennmedicine.upenn.edu

**Keywords:** Alzheimer’s disease, circulating cell free DNA, DNA methylation, epigenetics, artificial intelligence

## Abstract

Background: Despite extensive efforts, significant gaps remain in our understanding of Alzheimer’s disease (AD) pathophysiology. Novel approaches using circulating cell-free DNA (cfDNA) have the potential to revolutionize our understanding of neurodegenerative disorders. Methods: We performed DNA methylation profiling of cfDNA from AD patients and compared them to cognitively normal controls. Six Artificial Intelligence (AI) platforms were utilized for the diagnosis of AD while enrichment analysis was used to elucidate the pathogenesis of AD. Results: A total of 3684 CpGs were significantly (adj. *p*-value < 0.05) differentially methylated in AD versus controls. All six AI algorithms achieved high predictive accuracy (AUC = 0.949–0.998) in an independent test group. As an example, Deep Learning (DL) achieved an AUC (95% CI) = 0.99 (0.95–1.0), with 94.5% sensitivity and specificity. Conclusion: We describe numerous epigenetically altered genes which were previously reported to be differentially expressed in the brain of AD sufferers. Genes identified by AI to be the best predictors of AD were either known to be expressed in the brain or have been previously linked to AD. We highlight enrichment in the Calcium signaling pathway, Glutamatergic synapse, Hedgehog signaling pathway, Axon guidance and Olfactory transduction in AD sufferers. To the best of our knowledge, this is the first reported genome-wide DNA methylation study using cfDNA to detect AD.

## 1. Introduction

Alzheimer’s disease (AD) is the leading cause of severe dementia, however the etiological mechanisms of the disease have yet to be elucidated. The spectrum of putative AD pathophysiology is wide and expanding [1]. Mechanistic information on AD could yield clinical benefits. For example, information on disease pathogenesis could lead to the development of novel biomarkers and therapeutic targets. Given the long latency period and time course of AD, even in the absence of definitive treatment, therapies that slow disease progression or reduce the dementia burden can significantly improve the quality of life and yield substantial healthcare savings [2].

Epigenetic mechanisms regulate gene expression independent of DNA sequence changes [3]. DNA methylation is the most commonly studied epigenetic mechanism [4] and is known to play a significant role in AD pathogenesis while offering the future prospect of targeted correction [5]. Currently, circulating cfDNA, so-called ‘liquid biopsy’, is being used extensively in the study of cancer evolution [6,7], cardiomyocyte death [8] and as non-invasive biomarkers for transplant rejection [9,10,11]. Circulating nucleic acid levels were found to be elevated in the plasma of AD patients, the plasma of a transgenic mouse model of AD, and in the culture medium of cells treated with amyloid-β [12] raising interest in its potential as a source of AD biomarkers. Theoretically, neuronal, vascular, and inflammatory responses along with the anatomical and functional changes in the brain of AD sufferers, could be non-invasively monitored [13] in the future, given the fact that the DNA of cells from brain tissues contribute to the pool of circulating cfDNA.

There is intense research interest in the development of non-invasive blood-based biomarkers for AD. Potential advantages include reduced reliance on invasive or expensive diagnostic techniques such as lumbar puncture, positron emission tomography (PET) scans, and MRI techniques [14]. Artificial intelligence (AI) and machine learning (ML) approaches including deep learning (DL), offer distinct advantages in the analysis of the vast troves of biological data generated from omics experiments such as DNA-methylation [15,16,17,18]. AI and ML have been used to study metabolic pathways, drug-drug interaction and to predict significant markers in various diseases using multi-omics data [19,20,21]. Most of the reported studies in AD use neuroimaging data such as PET imaging and have demonstrated DL to be more accurate than all the platforms tested [22,23]. Systems biology techniques to include epigenetics, genomics, transcriptomics and metabolomics combined with both clinical and imaging data have been used to perform classification, biomarker identification, disease subtyping, disease progression prediction and drug repurposing [24]. With regard to transcriptomic studies and PM brain studies, AI analysis identifies candidate genes for AD [25]. Most of the reported AI based studies used invasive biomatrices such as PM brain tissue and the expensive imaging modalities. As such, there is a huge unmet need to develop non-invasive and inexpensive methods for diagnosing AD.

In this study, we performed methylation profiling of circulating cfDNA collected from individuals suffering from AD and compared them to cognitively healthy controls. Using AI analysis, we evaluated the accuracy of putative cytosine (CpG) epigenetic markers for AD diagnosis. Pathway analysis was used to further understand the molecular pathogenesis of AD.

## 2. Materials and Methods

The study was approved by the Human Investigation Committee of William Beaumont Hospital, Royal Oak, MI, USA (IRB#2017-214). Written consent was obtained from study participants or their legal representatives. A total of 52 subjects were prospectively recruited (26 AD cases and cognitively healthy 26 controls). The diagnosis of AD was based on existing clinical and laboratory criteria according to NINCDS-ADRDA [26]. Blood samples were collected from each subject in Streck Cell-Free DNA BCT^®^ tubes (Streck, La Vista, NE, USA). This minimizes further dilution and confounding from DNA that is released due to leukocyte lysis at the time of collection and during storage [27]. The samples were processed within 24 h of blood draw. For initial sample processing, specimens were centrifuged for 15 min at 3000× *g* and the plasma was aliquoted into 2.0 mL Eppendorf Safe-Lock micro-centrifuge tubes without disturbing the buffy coat, and subsequently stored at −80 °C for further processing [28]. The cfDNA was extracted twice for each sample from a total of 6 mL of plasma using the QIAamp circulating nucleic acid kit (Qiagen Cat # 55114) and a manual vacuum as per the manufacturer’s standardized protocol. We used a minimum of 8 ng/μL of DNA from each sample for sodium bisulfite conversion as measured using a nanodrop spectrophotometer. All samples were normalized on the Epic array plate using 300 ng of DNA per sample (8 ng × 37.5 μL). Per the manufacturer’s protocol, (EZ DNA Methylation kit), 200–500 ng of DNA provided optimal results. All samples were adjusted for the total volume of using water (45 μL) and 5 μL of M-dilution buffer was added and incubated at 37 °C for 15 min before adding the CT conversion reagent.

### 2.1. DNA Methylation Profiling

The extracted cfDNA was subjected to bisulfite conversion using the EZ DNA Methylation Kit (Zymo, Irvine, CA, USA) per the manufacturer’s instructions and the bisulfite converted DNA was eluted using 10 µL of elution buffer [29]. Following bisulfite conversion, we performed the Illumina Infinium MethylationEPIC BeadChip arrays for methylation profiling as per the manufacturer’s instructions. The vacuum dried BeadChips were imaged immediately on an Illumina iScan System (Illumina, Inc., San Diego, CA, USA).

### 2.2. Data Preparation

Missing values were detected and replaced by a small value which is the half of the minimum positive values in the original data. We made this assumption because most missing values are caused by the low abundance of CpG probes. Furthermore, the log value of each methylation level was centered by its mean and auto scaled by its standard deviation. The quantile normalization method was used to reduce sample-to-sample variation.

### 2.3. Statistical and Bioinformatic Analysis

All data analysis was performed using R version 4.1.1. Raw EPIC array data were processed using the package “minfi”. Noob normalization was used to normalize the signal. Outlier detection: Probe values not passing the detection threshold were marked as missing. Sex chromosome methylation probes were removed from the analysis to avoid gender specific methylation bias and to avoid the possible difficulties of having matched X and Y chromosome methylation markers caused by the epigenetic inactivation of one X chromosome in females [30]. The fraction of missing probe values were estimated for all samples and those with a fraction more than two standard deviations (95% confidence) away from the mean were deemed outliers. The K nearest neighbour algorithm with default parameters implemented in the “impute” package was used to impute missing values. Probes with variability higher than 0.01 across all samples were retained for further analysis. Immune cell type deconvolution was performed using the minfi package. Variance inflation: The proportion of granulocyte markers were identified as a strongly inflated covariate and correlated with other variables (Bcell, CD4T, CD8T, NK). After removal of the inflated covariate (granulocyte markers), other variables did not show any correlation with each other (Supplementary Figure S1).

The methylation beta values were transformed into M values and robust linear regression (M ~ b0 + b1 * ConditionAD + b2 * Age + b3 * GenderFemale + b4 * BMI + b5 * CD8T + b6 * CD4T + b7 * NK + b8 * Bcell + b9 * Mono + error) as implemented in the “limma” package was used to establish differentially methylated cytosines. The reported fold change (logFC) is the value of coefficient b1. We used a False Discovery Rate (FDR) correction (q < 0.05) as the significance threshold. In practice, we found that the q < 0.05 FDR adjusted value for EPIC arrays corresponds to roughly to a *p*-value = 1 × 10^−8^ [31]. Variance inflation:The regression model included concurrent medical disorders, age, gender and BMI as covariates, as well as the cell type proportions of CD8T, CD4T, NK, Bcell, and monocytes. As noted, hemolysis of these cell types can add to the apparent cfDNA pool in plasma. Other estimated immune cell type proportions were found to be colinear with the aforementioned ones and were not included in the model. A Fisher’s exact test comparing the number of significant hyper-methylated cytosines among all the significant cytosines to the total number of hyper-methylated cytosines among all interrogated cytosines was used to determine the overall trend towards hyper-methylation among significantly differentially methylated cytosines. Similarly, all cytosines were annotated with genomic and CpG island regions and enrichment of such regions with differentially modified cytosines was tested using Fisher’s exact test.

### 2.4. Enrichment Analysis

Pathway enrichment analysis was performed by annotating each EPIC array probe with the UCSC reference gene symbol. For each gene, we retained the CpG locus with the lowest overall *p*-value. The genes were subsequently ranked by negative log transformed *p*-values and passed to g:profiler service for enrichment analysis. Next, genes were ranked by the sign of fold change multiplied by negative log transformed *p*-value and passed to the gene set enrichment function implemented in the clusterProfiler package.

### 2.5. Artificial Intelligence/Deep Learning (AI/DL) Analysis

The detailed AI analysis is presented in our prior publications [18]. In brief, we used the overall CpG markers after normalization in AD subjects as compared to controls. We used DL and five other AI algorithms: Support Vector Machine (SVM), Generalized Linear Model (GLM), Prediction Analysis for Microarrays (PAM), Random Forest (RF), and Linear Discriminant Analysis (LDA) to perform classification and regression analysis [32]. More detailed information regarding the AI and DL models is available in the Supplementary Information section. We highlight the importance of the DL modeling. The main perception of DL is to learn data representations through increasing abstraction levels. Deep-learning methods are representation-learning methods with multiple levels of depiction, obtained by composing simple but non-linear modules that each transform the representation at one level (starting with the raw input) into a representation at a higher, slightly more abstract level. With the composition of enough such transformations, very complex functions can be learned. For classification tasks, higher layers of representation increases aspects of the input that are important for discrimination and suppress irrelevant variations. This type of hierarchical learning process is very powerful, as it allows a system to comprehend and learn complex representations directly from the raw data, making it useful in many disciplines.

Like other feed-forward artificial neural networks (ANNs), DL employs more than one hidden layer (y) that connects the input (x) and output layers (z) via a weight (W) matrix. The activation value of the hidden layer (y) can be calculated by sigmoid of the multiplication of the input sample x with the weight matrix W and bias b. The transpose of the weight matrix W and the bias b can then be used to construct the output (z) layer. The best set of the weight matrix W and bias b is expected to minimize the difference between the input layer (x) and the output layer (z).

Deep learning models are full of hyper-parameters and finding the best configuration for these parameters in such a high dimensional space is not a trivial challenge. The process of setting the hyper-parameters requires expertise and extensive trial and error. There are no simple and easy ways to set hyper-parameters. These hyper-parameters act as knobs that can be tweaked during the training of the model. For our model to provide the best result, we needed to find the optimal value of these hyper-parameters. We used a grid search to find out the best set of parameters. In grid search, we tried every possible configuration of the parameters. We first defined a grid on n dimensions, where each of these map to a hyper-parameter. For each dimension, we then defined the range of possible values. We searched for all the possible configurations and waited for the results to establish the best one. Finally, the grid search algorithm found the best set of parameters to give us the highest AUC result. The parameters and the ranges that we utilized in the models are as follows:

hyper_params <- list(
activation = c(“Rectifier”,“Tanh”),
hidden = list(c(100),c(200),c(10,10),c(20,20),c(50,50),c(30,30,30),c(25,25,25,25)),
input_dropout_ratio = c(0,0.05,0.1),
hidden_dropout_ratios = c(0.6,0.5,0.6,0.6),
l1 = seq(0,1e-4,1e-6),
l2 = seq(0,1e-4,1e-6),
train_samples_per_iteration = c(0,-2),
epochs = c(500),
momentum_start = c(0,0.5),
rho = c(0.5,0.99),
quantile_alpha = c(0,1),
huber_alpha = seq(0,1))

### 2.6. Validation

Ten-fold cross validation and bootstrapping methods were used for generating the model performance results. We randomly split the samples into 80% training and 20% testing sets. The 80/20 split is a common practice for moderate sizes which utilize ML-based approaches. We chose this ratio to have enough training samples to build a good model and sufficient testing samples to evaluate the model. We performed a 10-fold cross validation on the 80% training data during the model construction process and tested the model on the hold out 20%. We used the pROC R package to compute the area under the curve (AUC) of a receiver-operating characteristic (ROC) curve to assess the overall performance of the models. To avoid sampling bias, we repeated the above splitting process 100 times and calculated the average AUC on the hold out test sets. AUC measures the entire two-dimensional area underneath the entire ROC curve from (0,0) to (1,1). AUC provides an aggregate measure of performance across all possible classification thresholds. One way of interpreting AUC is as the probability that the model ranks a random positive example more highly than a random negative example. The AUC ranges in value from 0 to 1. A model whose predictions are 100% wrong has an AUC of 0.0; one whose predictions are 100% correct has an AUC of 1.0. 

The study patients were randomly separated into a ‘training’ group for the development of the predictive algorithm and an independent test or validation group to confirm the model’s performance. The intragenic (CpGs within gene region) and extragenic CpGs (CpGs outside of gene region) were considered separately in developing predictive models. Further details of the AI methods are provided in a Supplementary Methods Section. To compare performance methods, a pairwise Wilcoxon signed-rank test was used to estimate the statistical significance of the difference in performance between DL and other methods. We found out that the difference of DL and LDA is significant with the *p* value < 0.1.

### 2.7. Model Uncertainties

One of the uncertainties of the models is overfitting. To avoid overfitting in the DL model, we used three regularization parameters: L1, which increases model stability and causes many weights to become 0, and L2, which prevents weight enlargement. L1 lets only strong weights survive (constant pulling force towards zero), while L2 prevents any single weight from getting too big. Dropout [33] has recently been introduced as a powerful generalization technique, and is available as a parameter per layer, including the input layer. The key idea is to randomly drop units (along with their connections) from the neural network during training. This prevents units from co-adapting too much. The third parameter that we used for avoiding overfitting in DL modelling is input dropout ratio, which controls the amount of input layer neurons that are randomly dropped (set to zero), and controls overfitting with respect to the input data (useful for high-dimensional noisy data). For the other models, several parameters were used to tune the models and to overcome the overfitting problem: The number of trees for RF, classification cost for SVM and threshold amount for shrinking toward the centroid for PAM.

Other uncertainties of the model is to have clean data. To prepare the data for analysis we utilized several methods: missing values were detected and replaced a small value which is half of the minimum positive values in the original data. We made this assumption because most missing values are caused by low abundance metabolites. Furthermore, the log value of each methylation level was centered by its mean and auto scaled by its standard deviation. The quantile normalization method was used to reduce sample-to-sample variation.

To compare the performance methods, a pairwise Wilcoxon signed-rank test was used to estimate the statistical significance of the difference in performance between DL and other methods. We found out that only the difference of DL and LDA is significant with the *p* value < 0.1.

## 3. Results

**Figure 1:**
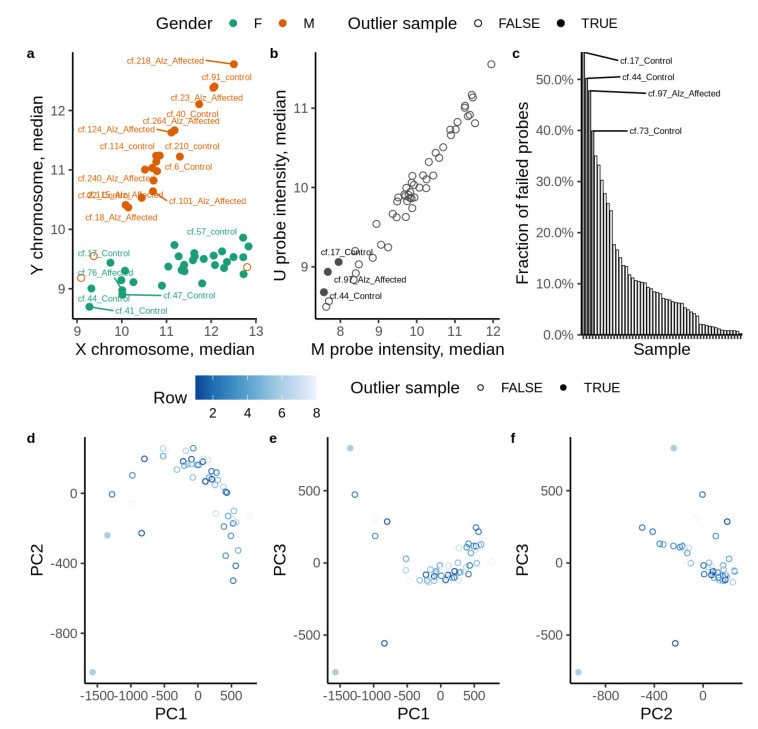
Detection of outliers in EPIC array methylation data. (**a**) Median signal intensity in sex chromosomes. (**b**) Median overall probe intensity. (**c**) Fraction of failed probes. Samples that deviate by more than 2 SD from the average fraction of failed probes are considered outliers. (**d**–**f**) Principal component analysis.

We evaluated genome-wide DNA methylation of circulating cfDNA from 26 people suffering from AD and compared them to 26 cognitively healthy controls. The study involved both familial AD cases and sporadic cases identified based on questionnaire and we did not genotype the samples. All control subjects were 60 years and above and age-matched to the AD group. All control subjects were cognitively tested using the standard battery of tests (MMSE scoring, SLUMS scoring and clinical dementia scoring; Supplementary Table S1). From closer inspection of the data, one AD subject and three controls were considered statistical outliers and removed from further analyses (Figure 1a–f). Clinical and demographic details are presented in Supplementary Table S1. The mean (SD) age was slightly higher in AD cases [82 (7)] versus controls [79 (9)], *p* = 0.01 and as such, all methylation changes were normalized for age. No other significant differences were noted for all other potential confounders to include gender (*p* = 0.52), ethnicity (*p* = 0.48), cardiovascular diseases or TBI (Supplementary Table S1). As expected, the Mini-Mental State Exam (MMSE) score was significantly lower for AD cases compared to controls: Mean (SD) = 20 (4) versus 29 (1), *p* < 0.001. Fifteen cases and 13 control samples were considered in the test group, while 10 cases and 10 controls were used in the training group.

### 3.1. Abundance of Significantly Methylated Cytosines

**Figure 2:**
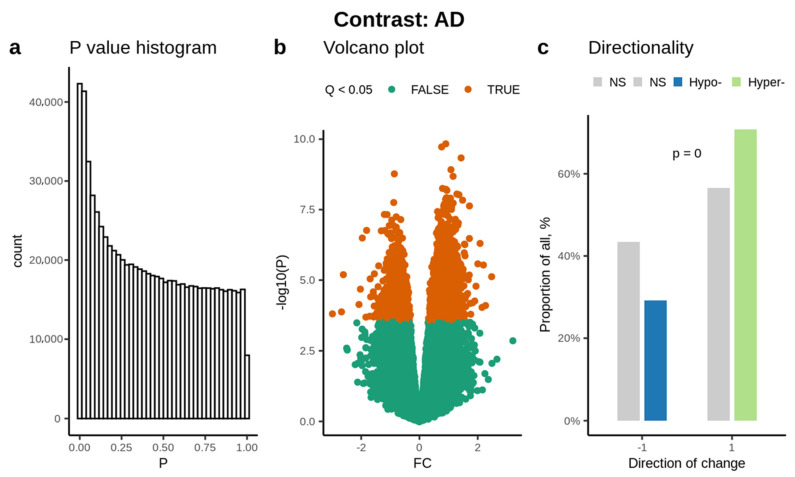
A linear model of DNA methylation in association with cell free circulating DNA in Alzheimer’s disease: Robust linear models fitted to the DNA methylation data using Age, Sex, NeuN proportion and Sentrix ID as covariates (**a**) Histogram based on *p*-value, showing CpGs being less than 0.05, (**b**) Volcano plot showing CpGs being less than 0.05 (orange colored nodes), (**c**) Overview of the methylation status of CpGs: the highest number of hyper-methylated CpGs (Green bar) were identified compared to hypo-methylated CpGs (Blue bar). The non-significant CpGs are presented using a grey scale.

Based on the *p*-value histogram, we identified a significant number of CpG methylation changes having a significance value less than 0.05 (Figure 2a), which is also reflected in the volcano plot (Figure 2b). 356,796 CpG probes passed the quality control. Overall, the study yielded a significantly higher number of hypermethylated CpGs (Figure 2c).

We identified a statistically significant change in methylation (adjusted *p* < 0.05) in a total of 3684 CpGs, among which 2729 CpGs were found to be hypermethylated and the remaining 955 CpGs were hypomethylated (Supplementary Table S2) in AD. We also identified 920 differentially methylated regions (DMRs) (adj. *p* < 0.05), among them, 854 DMRs were hypermethylated and the remaining 66 DMRs were hypomethylated (Supplementary Table S3).

### 3.2. Enrichment Analysis

Based on the enrichment of CpG regions, the CpGs on the islands were hypermethylated with a FDR *p* = 1.4 × 10^−137^. Based on the genomic regions, CpGs in the intergenic region were the most hypermethylated with FDR *p* = 5.1 × 10^−83^, followed by those in the promoter regions in AD cfDNA (FDR *p* = 8.8 × 10^−29^). Further details are provided in Supplementary Table S4 and Supplementary Figure S2a,b.

### 3.3. Disease and Functional Enrichment

**Figure 3:**
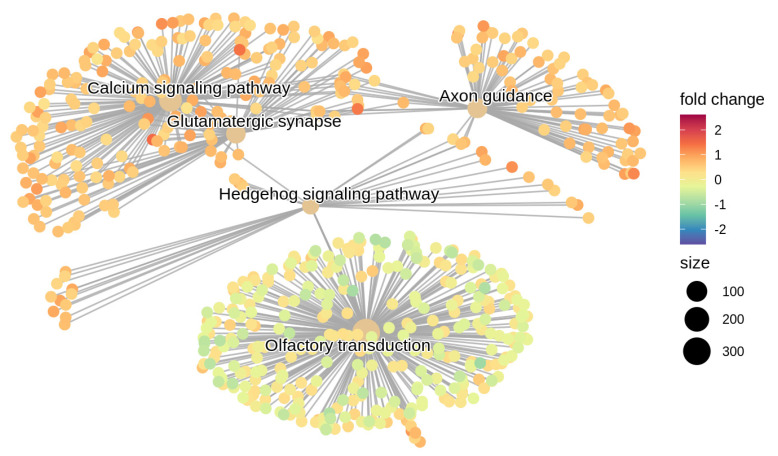
Visualization of gene network: Top 5 significant gene clusters are depicted- Calcium signaling pathway (q = 9.7 × 10^−5^), Glutamatergic synapse (q = 9.7 × 10^−5^), Hedgehog signaling pathway (q = 3.2 × 10^−4^), Axon guidance (q = 3.2 × 10^−4^) and Olfactory transduction (q = 4.4 × 10^−4^).

We used gene ontology analysis to identify biological processes and/or molecular functions associated with the differentially methylated genes. Analysis identified the Calcium signaling pathway (CpG set size = 227) (q = 9.77 × 10^−^^5^), Glutamatergic synapse (CpG set size = 109) (q = 9.77 × 10^−^^5^), Hedgehog signaling pathway (CpG set size = 52) (q = 0.00032), Axon guidance (CpG set size = 174) (q = 0.00032) and Olfactory transduction (CpG set size = 387) (q = 0.00044) as the top five perturbed networks. The cluster of genes encompassing these mechanisms are depicted in Figure 3. Detailed information of KEGG pathway identifiers, pathway description, statistical significance followed by the list of the enriched genes are provided in Supplementary Table S5.

### 3.4. AI Prediction of AD

A total of 262,046 intragenic and 94,750 extragenic CpG sites that passed QC were used for an unbiased AI analysis, immaterial of the *p*-value of the methylation change in AD cases. Training algorithms were developed using 15 AD cases and 13 controls and the performance of these algorithms was independently validated in an independent test group (10 AD cases and 10 controls).

The performance of the 20 intragenic CpG algorithms in the test group when a bootstrapping approach was used is shown in Table 1. The AI algorithms achieved excellent diagnostic performance in the independent test group AUC for the AI platforms (0.949−0.999). For example, in the independent test group, DL achieved an AUC (95% CI) = 0.998 (0.950−1.0), with 94.5% sensitivity and 94.5% specificity respectively, as shown in Table 1. The performance was close to that of the initial training data used to develop the algorithms, and is shown in Supplementary Table S6. The CpG predictors listed in decreasing order of contribution in each AI model are provided along with results from the training data in Supplementary Table S6. Similarly, excellent diagnostic performance was achieved in the independent test group using a 20 CpG intragenic algorithm-based 10-fold cross-validation (Table 2). The AUCs = 0.939–0.984 for the test group. For example, DL achieved an AUC (95% CI) = 0.984 (0.92−1.0), with 92.5% sensitivity and 93.5%specificity (Table 2). The performance in the training set used to develop the 20 marker predictive algorithms with 10-fold cross-validation is shown in Supplementary Table S7. The CpG predictors in each model are presented in decreasing order of contribution to prediction along with results in its training data set, and are provided in Supplementary Table S7.

We also evaluated the performance of 20 variables using extragenic only CpG markers with bootstrapping. Overall, the AI platforms achieved an AUC = 0.949–0.999 in the independent test group (Table 3). DL achieved an AUC (95% CI) = 0.999 (0.95−1.0), with 94.5% sensitivity and 94.5% specificity (Table 3). The performance in the training group used to develop the algorithms is shown for comparison (Supplementary Table S8). The CpG predictors in decreasing order of contribution in each model are provided along with the associated training results in Supplementary Table S8. Finally, we also evaluated the performance of more parsimonious models using only 5 CpGs for intragenic CpG markers using bootstrapping. The performance of the different AI 5-marker algorithms is shown for the test group in Supplementary Table S9 and for the related training group used to develop the models for the sake of comparison is shown in Supplementary Table S10. The performance for the test group was slightly lower than for the 20-marker algorithm for the 6 AI platforms (AUC = 0.899−0.923). For example, the DL algorithm achieved an AUC (95% CI) = 0.92 (0.81–1.0), with 92.5% sensitivity and 93.5% specificity.

Our study focused on circulating cfDNA and therefore we were unable to evaluate gene expression. We did however investigate whether there was a possible correlation between our circulating cfDNA methylation analysis and previously published brain transcriptomic studies. O’Connell et al. (2020) [34] collated and performed a bioinformatic analysis of published studies that evaluated mRNA expression data. Using a total of 12,000 human specimens, they evaluated 17,000 protein-coding genes and determined their feasibility as blood biomarkers for brain damage. Genes were considered and ranked as possible biomarkers for brain injury based on the following criteria: (i) enrichment in brain tissue compared to non-neuronal tissue, (ii) abundantly expressed in brain and (iii) had low expression variability across various brain regions. Of the top 100 ‘brain biomarker’ genes identified by O’Connell et al. (2020) [34], we found 16 genes (16%) that were differentially methylated (adjusted *p* < 0.05) in our study. They include, *C11orf87, FBXL16, GABRA5, GNG13, GPM6A, GRM4, HPCA, KCNN1, KLHL1, LRTM2, NR2E1, SLC17A7, SLC1A2, SNCB, SOX1* and *SYNPR*. The primary neurological cell type of preferential expression of these is shown in Supplementary Figure S3. Similarly, Shigemizu et al. (2020) [35] evaluated mRNA expression using blood based transcriptomic analysis in AD versus controls. They identified 846 significantly differentially expressed genes. When we correlated those with our results, we found 102 genes (12%) that were also differentially methylated in our study.

## 4. Discussion

Circulating cfDNA is classically released into the bloodstream from damaged or dead tissues into the brain [36]. Using DNA-methylation analysis of circulating cfDNA, we report extensive epigenetic modification in cytosine nucleotides in genes from individuals diagnosed with AD as compared to cognitively healthy control subjects. Multiple different algorithms were evaluated using six different AI platforms and different analytic approaches. Combining AI analysis with DNA methylation data from circulating cfDNA, we achieved excellent diagnostic accuracy for AD. This was true when either intra- and extra-genic CpG markers were considered. The observed diagnostic accuracy was sustained using different analytic approaches (e.g., cross-validation and bootstrapping) and with the use of parsimonious models consisting of 5 predictive CpGs. An important objective of our study was to use cfDNA to further elucidate the molecular mechanisms of AD. We identified epigenetic changes in molecular pathways previously linked to neurological disease, and thus are congruent with our current understanding of AD.

We found increased hypermethylation of CpGs in cfDNA from AD patients across the genome as compared to controls (Figure 2c). The gene promoter and 5′UTR regions were increasingly hypermethylated as opposed to hypomethylated (Supplementary Table S4) in AD. Hypermethylation classically regulates the genome by silencing gene promoters, silencing or at least downregulating (partial activity) the enhancers, and through the control of non-coding RNA genes [37]. Overall, these results suggest the possible downregulation of gene expression in association with AD.

Here we review some of the genes that were found to be significantly differentially methylated and provide information on their known or putative roles in neuronal function and AD. *KDM2A* was the most significantly differentially methylated (hyper-methylated) gene at the Transcription Start Site 1500 (TSS1500; adj. *p* = 7.45 × 10^−5^) and is involved in histone demethylase activity. Essentially, it recruits HP1 and establishes H3K9 and CpG methylation to form mature heterochromatin and regulates complex nucleosome binding mechanisms. Disrupted nucleosome binding results in transcriptional deregulation and genomic instability [38]. This mechanism was reported to be disrupted in synaptic genes of AD affected brains [39,40]. The second most significantly differentially methylated gene was *ZNF529,* which was hyper-methylated at TSS1500 and the 5′ UTR (adjusted *p* = 7.45 × 10^−5^). While this gene has not previously reported to be associated with neurodegenerative disorders, blocking its activity results in increased low-density lipoprotein (LDL) receptor expression and with increased cholesterol (LDL-c) uptake by cells in association with cardiovascular diseases (CVD) [41]. It is notable that CVD and LDL-c are both significant AD risk modifiers [42]. The next gene found to undergo significant methylation change was *HOXD13*. This gene was hyper-methylated on exon 1 and is involved in regulating neuronal stemness [43]. The role of this gene in AD pathogenesis is yet to be explored.

AI algorithms are increasingly being utilized to build accurate disease predictors based on big data from omics experiments [44]. As noted, we developed excellent AD diagnostic models using multiple platforms (e.g., DL, SVM, GLM, PAM and RF) that were validated in an independent test group. The AI algorithms rank the contribution of markers (in decreasing order of contribution (Table 1, Table 2 and Table 3 and Supplementary Tables S6–S10). Based on AI ranking, we were able to identify 5 CpG markers that appeared to be the best AD predictors across the different platforms. Five CpGs: cg19760734 (*TACC1*), cg05876416 (*FAM173B*), cg00234736 (*ELMO1*), cg21243612 (*C9orf6*), and cg24040188 (*RBBP8*) consistently appeared among the four AI algorithms (SVM, PAM, RF and DL) for AD detection (Table 1). We reviewed the literature to determine the potential biological relevance of these genes in relation to AD. *TACC1, FAM173B, C9orf6* and *RBBP8* are expressed in various regions of the brain according to “The Genotype-Tissue Expression (GTEx)” portal [45]. Further work is needed to ascertain their possible roles in AD. In contrast, *ELMO1* has been linked to AD. A knock down of *ELMO1* inhibits neurite outgrowth and deactivates Rac1 and Rac1 mediated neurite outgrowth leading to age-dependent neurodegeneration and AD development [46,47]. To the best of our knowledge, no prior study has reported on AD detection based on cfDNA. However, a study using brain epigenetic analysis identified kinases associated with AD [48]. DNA methylation analysis based on brain tissue [49] has achieved good predictive accuracy with an AUC of >0.79 [50] but not an excellent predictive accuracy. Another study with the same dataset [49] along with an additional dataset [51] also did not reach an excellent predictive accuracy [52]. A computational approach aided by supervised ML was used to identify predictive CpGs from microarray-based Epigenome Wide Association studies (EWAS) for six traits associated with AD. Postmortem brain tissue was used to generate CpG methylation data using the Illumina-450 array. These traits were beta-amyloid accumulation, neurofibrillary tangles (NFTs), Braak staging, Consortium to establish a Registry of Alzheimer’s Disease (CERAD) score, global pathology and cognitive trajectory study using epigenetic analysis of brain tissue [48]. CpG methylation had AUCs = 0.850−0.962 for trait detection. The AUC for the detection of NFT, a hallmark of AD, was equal to 0.962. A study of DNA methylation in brain tissue [49] reported good predictive accuracy, with an AUC of >0.79 for AD detection [50]. A subsequent in silico publication combining two prior datasets [49] in which methylation profiling of the superior temporal gyrus of AD cases and controls was used [51]. Random Forrest feature selection evaluated the diagnostic performance of a 10 CpG marker algorithm for AD detection. After 10-fold CV the decision tree (DT), SVM, and RF models achieved an AUC of 89.6, 75.8 and 92.7, respectively. Overall, these studies indicate a strong correlation between CpG methylation with AD in general, cognitive performance in AD and marquee brain histologic changes used to define AD such as NFT.

### Disease and Functional Enrichment

Beyond the possible role of individual genes, we evaluated gene networks to further our understanding of AD. We found significant over-representation of gene pathways linked to neurological disease: Calcium signaling pathway, Glutamatergic synapse, Hedgehog signaling pathway, Axon guidance, and Olfactory transduction.

Calcium signaling pathway: Calcium is an important signaling ion, and the disrupted calcium homeostasis is linked to amyloid plaque (Aβ) formation in AD. Calcium signaling is linked to the Calcium/calmodulin-dependent kinases MAPK/ERKs and the CREB cycle which regulates the homeostasis in AD [53,54,55]. In AD, the amyloidogenic pathway remodels neuronal Ca^2+^ signaling, leading to enhanced cellular entry of Ca^2+^ through ryanodine receptors [56]. Disrupted cellular calcium can induce synaptic deficits that promote the accumulation of Aβ and neurofibrillary tangles [57], both of which are marquee pathological features of AD. The gene *CACNA1C* displayed altered methylation in 5 CpG loci (3 hyper- and 2 hypo-methylated). The interaction between *RYR3* and *CACNA1C* is crucial in terms of AD pathogenesis. Both genes are involved in modulating Aβ load and increasing intracellular calcium levels [58]. *MYLK* (hypermethylated CpGs in AD as reported herein) codes for myosin light chain kinase (MLCK). MLCK is involved in hippocampal neuronal microfilament damage in hyperglycemia. Chronic hyperglycemia induces irregularities in nuclear shape, induces shrinking of synapses and thus damages the neuronal microfilament [59]. Hyperglycemia is an established risk factor for AD development [59].

Glutamatergic synapse: Excitatory glutamatergic neurotransmission is essential for synaptic plasticity and neuronal survival. This type of neurotransmission occurs via the N-methyl-d-aspartate receptor (NMDAR) [60]. Synaptic NMDAR supports plasticity and promotes cell survival while extrasynaptic NMDAR promotes excitotoxicity which leads to cell death and neurodegeneration, a hallmark of AD [60]. Differentially methylated genes involved in the Glutamatergic synapse include the *PPP3CB gene. PPP3CB* codes for protein phosphatases that reverse the activity of protein kinases, which are important in the process of tau and amyloid-β accumulation [61]. *PPP3CB* was previously reported to be linked to long-term memory potentiation in AD [62]. We also identified epigenetic changes in genes from the solute carrier (SLC) superfamily of solute carrier transporters. The SLC superfamily participates in the uptake of small molecules into cells [63]. We identified 86 differentially methylated SLC superfamily genes in our study, five of which (*SLC8A3, SLC1A2*, *SLC1A6, SLC17A7, SLC24A4*) were found to be enriched in significant signaling pathways in this study. *SLC8A3* is involved in Calcium signaling, and along with *SLC1A2*, *SLC1A6,* and *SLC17A7* is known to participate in glutamatergic synapses, while *SLC24A4* is involved in olfactory transduction. In the brain, SLC family transporters are important for returning synaptic neurotransmitters to the presynaptic neurons [63,64]. Altered expression of these genes can lead to synaptic dysfunction, an important feature of AD pathogenesis [65].

Hedgehog signaling pathway: The Sonic hedgehog (SHH) signaling pathway is involved in neurogenesis, neural patterning and cell survival during nervous system development [66,67]. SHH signaling requires intact primary cilia in brain cells and fails with structurally disrupted cilia. Elevated Aβ peptide levels that result in plaque formation disrupt the cilial structure and thus inhibit SHH signaling. Human ciliary disease results in cognitive impairment, a feature of AD [67]. We found epigenetic changes in genes involved in the SHH signaling pathway, including the *CDON CUL3* and *GLI3* genes. The *CDON* gene may participate in the generation of neurons and in nervous system development [68]. The *CUL3* gene is one of the ubiquitin ligase genes and it was found to be downregulated in various brain regions in AD subjects [69]. We found hypermethylation of this gene which is consistent with the down-regulation of gene expression. *GLI3* is a gene that we found to be hypermethylated and which has previously been linked to language dysfunction in AD [70].

Axon guidance: Axonal guidance is a neurodevelopmental process in which the axons are directed to their target neurons. The molecules involved in axon guidance also play key roles in immune and inflammatory responses in the nervous system [71]. Several of the genes involved in axon guidance were also differentially methylated in our study. BMP7 is involved in Axon guidance [72] and in the recovery of cardiac function after myocardial infarction [73]. We found hypomethylation of this gene in AD. BMP7 is a candidate gene for vascular diseases [74]. The gene variants of BMP7 stimulate inflammation and are associated with acute myocardial infarction and AD [75]. The other gene identified in axon guidance is *MYL9*, which codes for the myosin light chain. Biologically, it interacts with *NMDAR,* which regulates synaptic plasticity and thereby regulates neurons in the hippocampus [76,77]. *SEMA6D* is a cardiac expressed gene that codes for semaphorins. *SEMA6D* interacts with *TREM2*, which is a gene that is involved in axonal growth in AD, and has been linked to AD pathogenesis [78].

Olfactory transduction: The olfactory neurons are thought to provide an entry portal into the brain for external substances believed to be involved in the pathophysiology of major neurodegenerative disorders such as AD and Parkinson’s disease. Diminution of the sense of smell is a common non-specific feature of early stage Parkinson’s disease [79] and also AD. *NCALD* codes for Neurocalcin delta, which is a neuronal calcium sensor [80]. Complete loss of function of the gene is believed to impair neurogenesis, and reduced expression in the brains of AD subjects has been reported [80,81].

One limitation of our study is that given the focus on circulating cfDNA, we were unable to perform expression analysis specific to the DNA. As brain cells also contribute to the circulating cfDNA pool, we investigated whether there was a possible correlation between our methylation findings and published brain transcriptomic studies. Of the top 100 ‘biomarker’ genes indicating neurological damage identified by O’Connell et al. (2020) [34], we found that 16% of these damage genes, which are known to be differentially expressed in the brain, are also be differentially methylated (adjusted *p* < 0.05) in circulating cfDNA in AD cases. Furthermore, based on specific biomarker enrichment analysis, we found astrocyte and neuronal coding genes to be significantly differentially methylated along with other genes in which the cell type in which the gene is preferentially expressed is unknown (Supplementary Figure S3). The differentially methylated astrocyte coding genes found to be enriched in AD cases were *SLC1A2* (one CpG hypomethylated and two hypermethylated) and *GPM6A* (1 CpG hypermethylated). The differentially methylated neuron enriched genes were *FBXL16, HPCA*, *SNCB*, and *SYNPR*. All these neuronal associated CpGs were hypermethylated in our study. For the remaining 12 differentially methylated genes, the origin of the brain cells in which they are differentially expressed is listed as “currently unknown” [34]. We also compared our significantly differentially methylated genes with single cell transcriptomic analysis in AD using prefrontal cortex tissue [82]. We found 116 differential methylated genes showing differential expression in astrocytes, and 21 differential methylated genes showed similar differential expression in microglial cells based on an adjusted *p*-value <0.05 in both of the data sets. Overall, these findings suggest a possible correlation between gene expression in the brain and the circulating cfDNA methylation markers. Finally, we compared our significantly differentially methylated genes with those reported by Shigemizu et al. (2020) [35] and found 12% of the genes to be differentially expressed in the blood. Based on this, we predict that higher concentrations of cfDNA may derive from the brain of AD subjects due to neuronal damage.

Identifying the organ of origin has been shown to be feasible [83], and could prove to be valuable in future studies. We have the limitation of using a relatively modest sample size in this study; despite this limitation, we did demonstrate strong diagnostic performances. Validation in larger cohorts is required, as much larger studies using cfDNA help to further elucidate the etiopathogenesis of AD while also developing novel, diagnostic biomarker panels.

## 5. Conclusions

We report significant genome-wide methylation changes in circulating cfDNA from AD subjects. Using multiple AI techniques and both intragenic or extragenic CpG methylation markers in an independent test or validation group, we report excellent diagnostic accuracy (AUCs of ≥0.9) for AD. Intriguing and plausible pathogenic information on AD development was also generated. Multiple genes that were epigenetically altered in AD in our study were previously known or linked to the control of synaptic activity, neuronal stemness and age-dependent neurodegeneration. A substantial number of genes that are highly ranked as plausible markers for brain damage based on their differential expression in the brain were also found to be differentially methylated in circulating cfDNA. Finally, using pathway analysis, we found epigenetic dysregulation of gene networks involved in neurotransmission, synaptic plasticity, cell survival, learning and function of memory. Our findings provide the basis and justification for larger patient studies.

## Figures and Tables

**Table 1:** Artificial Intelligence and circulating cfDNA prediction for the Alzheimer’s disease intragenic CpGs (20 Variables Bootstrapping–Independent Test group).

	SVM	GLM	PAM	RF	LDA	DL
AUC 95% CI	0.9760 (0.8900–1)	0.9773 (0.8500–1)	0.9886 (0.9000–1)	0.9874 (0.9000–1)	0.9493 (0.9500–1)	0.9988 (0.9500–1)
Sensitivity	0.9200	0.9200	0.9200	0.9300	0.9350	0.9450
Specificity	0.9220	0.9090	0.9080	0.9100	0.9250	0.9450

Support Vector Machine (SVM), Generalized Linear Model (GLM), Prediction Analysis for Microarrays (PAM), Random Forest (RF), Linear Discriminant Analysis (LDA) and Deep Learning (DL).

**Table 2:** Artificial Intelligence and circulating cfDNA prediction for the Alzheimer’s disease intragenic CpGs (20 Variables Cross Validation–Independent Test group).

	SVM	GLM	PAM	RF	LDA	DL
AUC 95% CI	0.9700 (0.8700–1)	0.9673 (0.8800–1)	0.9786 (0.8800–1)	0.9774 (0.8800–1)	0.9393 (0.8700–1)	0.9840 (0.9250–1)
Sensitivity	0.9100	0.9100	0.9100	0.9200	0.9250	0.9250
Specificity	0.9120	0.8990	0.8980	0.9000	0.9150	0.9350

Support Vector Machine (SVM), Generalized Linear Model (GLM), Prediction Analysis for Microarrays (PAM), Random Forest (RF), Linear Discriminant Analysis (LDA) and Deep Learning (DL).

**Table 3:** Artificial Intelligence and circulating cfDNA prediction for the Alzheimer’s disease Extragenic CpGs (20 Variables Bootstrapping–Independent Test group).

	SVM	GLM	PAM	RF	LDA	DL
AUC 95% CI	0.9770 (0.8900–1)	0.9744 (0.8500–1)	0.9856 (0.9000–1)	0.9860 (0.9000–1)	0.9488 (0.9500–1)	0.9988 (0.9500–1)
Sensitivity	0.9200	0.9200	0.9200	0.9300	0.9350	0.9450
Specificity	0.9220	0.9090	0.9080	0.9100	0.9250	0.9450

Support Vector Machine (SVM), Generalized Linear Model (GLM), Prediction Analysis for Microarrays (PAM), Random Forest (RF), Linear Discriminant Analysis (LDA) and Deep Learning (DL).

## Data Availability

The data that support the findings of this study are available on request from the corresponding authors.

## Supplementary Materials

The following are available online at https://www.mdpi.com/article/10.3390/cells11111744/s1, Supplementary Figure S1: Variance inflation analysis using all specified covariates (Full) and after removal of inflated covariates (Reduced); Supplementary Figure S2: (a) Enrichment of CpGs in various regions of the genome (CpG islands) and (b) the enrichment of genomic features including intergenic and within gene regions; Supplementary Figure S3: Enrichment of differentially methylated genes in neurological damage biomarkers panel. The correlation analysis used O’Connell et al., (2020) study with about 12,000 human subjects’ mRNA expression data; Supplementary Table S1: Comparison of demographics and clinical characteristics: Alzheimer’s disease cases vs. normal controls; Supplementary Table S2: Significantly differentially methylated cytosine loci (CpGs) based on adjusted *p*-value for circulating cfDNA in Alzheimer’s disease.; Supplementary Table S3: Significantly differentially methylated regions (DMRs) based on adj. *p*-value in association with cfDNA in Alzheimer’s disease; Supplementary Table S4: DNA methylation changes in circulating cell free circulating DNA in Alzheimer’s disease in different genomic regions Supplementary Table S5: List of KEGG based molecular mechanisms enriched with differentially methylated genes are provided. The table details the KEGG pathway identifiers, CpG set size, statistical significance, core enriched genes and the differentially methylated genes of top 5 significant pathways are listed; Supplementary Table S6: Artificial Intelligence and circulating cfDNA methylation for prediction of Alzheimer’s disease using intragenic CpGs (20 Variables Bootstrapping–Training group); Supplementary Table S7: Artificial Intelligence and circulating cfDNA methylation for prediction of Alzheimer’s disease using intragenic CpGs (20 Variables Cross Validation–Training group); Supplementary Table S8: Artificial Intelligence and circulating cfDNA for prediction of the Alzheimer’s disease -extragenic CpGs (20 Variables Bootstrapping–Training group); Supplementary Table S9: Artificial Intelligence and circulating cfDNA prediction of Alzheimer’s disease -intragenic CpGs (5 Variables Bootstrapping–Test group); Supplementary Table S10: Artificial Intelligence and circulating cfDNA for the prediction of Alzheimer’s disease -intragenic CpGs (5 Variables Bootstrapping–Training group) [18,32,84,85,86,87,88,89].
