# Supplementary material for: Artificial Intelligence and Circulating Cell-Free DNA Methylation Profiling: Mechanism and Detection of Alzheimer’s Disease
Source: Cells. 2022 May 25;11(11):1744. doi: 10.3390/cells11111744 (PMC9179874; doi:10.3390/cells11111744)
Supplement: Supplementary file 1 [file cells-11-01744-s001.zip › Supp Table S1.pdf]

**Supplemental Table S1:** Comparison of demographics and clinical characteristics: Alzheimer's disease cases vs. normal controls.

| Parameter | Cases | Controls | q-value (FDR) |
|---|---|---|---|
| **Number of patients** | 26 | 26 | - |
| **Age [Mean (Standard deviation)]** | 82.45 (7.11) | 79.26 (9.63) | 0.01 (W) |
| **Gender (%)** | | | |
| Females | 50 | 65.38 | 0.52 (W) |
| Males | 42.30 | 34.61 | |
| Data unavailable | 7.69 | 0 | |
| **Race (%)** | | | |
| Non-Hispanic | 92.30 | 88.46 | 0.48 (W) |
| Hispanic | 0 | 7.69 | |
| Not reported | 7.69 | 3.84 | |
| **MMSE Score [Mean (Standard deviation)]** | 20.09 (4.74) | 28.92 (1.07) | < 0.0001 (W) |
| **Stroke (%)** | | | |
| Yes | 7.69 | 7.69 | 0.11 (W) |
| No | 80.76 | 88.46 | |
| Data unavailable | 11.53 | 3.84 | |
| **Hyperlipidemia (%)** | | | |
| Yes | 73.07 | 65.38 | 0.52 (W) |
| No | 19.23 | 30.76 | |
| Data unavailable | 7.69 | 3.84 | |
| **Hypertension (%)** | | | |
| Yes | 65.38 | 61.53 | 0.40 (W) |
| No | 26.92 | 34.61 | |
| Data unavailable | 7.69 | 3.84 | |
| **Diabetes (%)** | | | |
| Yes | 19.23 | 26.92 | 0.14 (W) |
| No | 73.07 | 69.23 | |
| Data unavailable | 7.69 | 3.84 | |
| **Traumatic Brain Injury (TBI) (%)** | | | |
| Yes | 23.07 | 3.84 | 0.52 (W) |
| No | 69.23 | 92.30 | |
| Data unavailable | 7.69 | 3.84 | |
| **BMI [Mean (Standard deviation)]** | 26.43 (4.21) | 25.81 (5.03) | 0.40 (W) |

W- Wilcoxon Mann Whitney test
