# Supplementary material for: Artificial Intelligence and Circulating Cell-Free DNA Methylation Profiling: Mechanism and Detection of Alzheimer’s Disease
Source: Cells. 2022 May 25;11(11):1744. doi: 10.3390/cells11111744 (PMC9179874; doi:10.3390/cells11111744)
Supplement: Supplementary file 1 [file cells-11-01744-s001.zip › Supp Table S10.pdf]

Supplemental Table S10: Artificial Intelligence and circulating cfDNA prediction for the Alzheimer's disease intragenic CpGs (5 Variables Bootstrapping – Training group)

	SVM	GLM	PAM	RF	LDA	DL
AUC<br>95% CI	0.9230<br>(0.8000-1)	0.9150<br>(0.8000-1)	0.9190<br>(0.8000-1)	0.9120<br>(0.8000-1)	0.9011<br>(0.8000-1)	0.9288<br>(0.8220-1)
Sensitivity	0.9100	0.9200	0.9200	0.9200	0.9250	0.9450
Specificity	0.9220	0.9090	0.9080	0.9100	0.9250	0.9450

Support Vector Machine (SVM), Generalized Linear Model (GLM), Prediction Analysis for Microarrays (PAM), Random Forest (RF), Linear Discriminant Analysis (LDA) and Deep Learning (DL)

Important predictors in decreasing order of contribution:
SVM: cg19760734, cg05876416, cg00234736, cg21243612, cg24040188
GLM: cg19038282, cg20573828, cg23301353, cg21317441, cg23962555
PAM: cg19760734, cg05876416, cg00234736, cg21243612, cg24040188
RF: cg19760734, cg05876416, cg14523095, cg10504568, cg00234736
LDA: cg14523095, cg10504568, cg08623971, cg16166011, cgg07748806
DL: cg19760734, cg05876416, cg00234736, cg21243612, cg24040188
