# Supplementary material for: Artificial Intelligence and Circulating Cell-Free DNA Methylation Profiling: Mechanism and Detection of Alzheimer’s Disease
Source: Cells. 2022 May 25;11(11):1744. doi: 10.3390/cells11111744 (PMC9179874; doi:10.3390/cells11111744)
Supplement: Supplementary file 1 [file cells-11-01744-s001.zip › Supp Table S6.pdf]

Supplemental Table S6: Artificial Intelligence and circulating cfDNA prediction for the Alzheimer's disease intragenic CpGs (20 Variables Bootstrapping – Training group)

	SVM	GLM	PAM	RF	LDA	DL
AUC<br>95% CI	0.9800<br>(0.9000-1)	0.9875<br>(0.9000-1)	0.9950<br>(0.9000-1)	0.9930<br>(0.9000-1)	0.9575<br>(0.9500-1)	1.0000<br>(0.9600-1)
Sensitivity	0.9300	0.9300	0.9300	0.9300	0.9350	0.9550
Specificity	0.9320	0.9120	0.9180	0.9200	0.9250	0.9550

Support Vector Machine (SVM), Generalized Linear Model (GLM), Prediction Analysis for Microarrays (PAM), Random Forest (RF), Linear Discriminant Analysis (LDA) and Deep Learning (DL)

Important predictors in decreasing order of contribution:

SVM: cg19760734, cg05876416, cg00234736, cg21243612, cg24040188, cg17674653, cg21942438, cg18322696, cg11748187, cg00266619, cg14523095, cg10504568, cg08623971, cg16166011, cgg07748806, cg04863005, cg00360534, cg07018367, cg23313274, cg23736989
GLM: cg19038282, cg20573828, cg23301353, cg21317441, cg23962555, cg06981876, cg12477067, cg14197110, cg16198754, cg07674600, cg25645008, cg05210497, cg04955826, cg14139646, cg19144827, cg06183001, cg12647020, cg00249383, cg02308140, cg24744710
PAM: cg19760734, cg05876416, cg00234736, cg21243612, cg24040188, cg14523095, cg10504568, cg08623971, cg16166011, cgg07748806, cg17674653, cg21942438, cg18322696, cg11748187, cg00266619, cg04863005, cg00360534, cg07018367, cg23313274, cg23736989
RF: cg19760734, cg05876416, cg14523095, cg10504568, cg00234736, cg21243612, cg08623971, cg16166011, cg24040188, cg17674653, cgg07748806, cg04863005, cg21942438, cg18322696, cg00360534, cg07018367, cg11748187, cg00266619, cg06981876, cg12477067
LDA: cg14523095, cg10504568, cg08623971, cg16166011, cgg07748806, cg04863005, cg00360534, cg07018367, cg23313274, cg23736989, cg06183001, cg12647020, cg00249383, cg02308140, cg24744710, cg06981876, cg12477067, cg14197110, cg16198754, cg07674600
DL: cg19760734, cg05876416, cg00234736, cg21243612, cg24040188, cg17674653, cg21942438, cg18322696, cg11748187, cg00266619, cg25645008, cg05210497, cg04955826, cg14139646, cg19144827, cg19038282, cg20573828, cg23301353, cg21317441, cg23962555
