# Supplementary material for: Artificial Intelligence and Circulating Cell-Free DNA Methylation Profiling: Mechanism and Detection of Alzheimer’s Disease
Source: Cells. 2022 May 25;11(11):1744. doi: 10.3390/cells11111744 (PMC9179874; doi:10.3390/cells11111744)
Supplement: Supplementary file 1 [file cells-11-01744-s001.zip › Supp Table S7.pdf]

Supplemental Table S7: Artificial Intelligence and circulating cfDNA prediction for the Alzheimer's disease intragenic CpGs (20 Variables Cross Validation – Training group)

	SVM	GLM	PAM	RF	LDA	DL
AUC<br>95% CI	0.9740<br>(0.8800-1)	0.9690<br>(0.8900-1)	0.9890<br>(0.8900-1)	0.9854<br>(0.8800-1)	0.9493<br>(0.8800-1)	0.9890<br>(0.9300-1)
Sensitivity	0.9200	0.9200	0.9200	0.9200	0.9250	0.9350
Specificity	0.9220	0.9090	0.9080	0.9200	0.9250	0.9350

Support Vector Machine (SVM), Generalized Linear Model (GLM), Prediction Analysis for Microarrays (PAM), Random Forest (RF), Linear Discriminant Analysis (LDA) and Deep Learning (DL)

Important predictors in order:
SVM: cg19760734, cg05876416, cg00234736, cg21243612, cg24040188, cg17674653, cg21942438, cg18322696, cg11748187, cg00266619, cg14523095, cg10504568, cg08623971, cg16166011, cgg07748806, cg04863005, cg00360534, cg07018367, cg23313274, cg23736989
GLM: cg19038282, cg20573828, cg23301353, cg21317441, cg23962555, cg06981876, cg12477067, cg14197110, cg16198754, cg07674600, cg25645008, cg05210497, cg04955826, cg14139646, cg19144827, cg06183001, cg12647020, cg00249383, cg02308140, cg24744710
PAM: cg19760734, cg05876416, cg00234736, cg21243612, cg24040188, cg14523095, cg10504568, cg08623971, cg16166011, cgg07748806, cg17674653, cg21942438, cg18322696, cg11748187, cg00266619, cg04863005, cg00360534, cg07018367, cg23313274, cg23736989
RF: cg19760734, cg05876416, cg14523095, cg10504568, cg00234736, cg21243612, cg08623971, cg16166011, cg24040188, cg17674653, cgg07748806, cg04863005, cg21942438, cg18322696, cg00360534, cg07018367, cg11748187, cg00266619, cg06981876, cg12477067
LDA: cg14523095, cg10504568, cg08623971, cg16166011, cgg07748806, cg04863005, cg00360534, cg07018367, cg23313274, cg23736989, cg06183001, cg12647020, cg00249383, cg02308140, cg24744710, cg06981876, cg12477067, cg14197110, cg16198754, cg07674600
DL: cg19760734, cg05876416, cg00234736, cg21243612, cg24040188, cg17674653, cg21942438, cg18322696, cg11748187, cg00266619, cg25645008, cg05210497, cg04955826, cg14139646, cg19144827, cg19038282, cg20573828, cg23301353, cg21317441, cg23962555
