# Supplementary material for: Artificial Intelligence and Circulating Cell-Free DNA Methylation Profiling: Mechanism and Detection of Alzheimer’s Disease
Source: Cells. 2022 May 25;11(11):1744. doi: 10.3390/cells11111744 (PMC9179874; doi:10.3390/cells11111744)
Supplement: Supplementary file 1 [file cells-11-01744-s001.zip › Supplemental Methods.pdf]

Supplemental Methods:

Artificial Intelligence (AI) Analysis:

Random Forest (RF) is a supervised learning algorithm for classification, regression, and other functions. It is supervised in the respect that the function is inferred from initially labelled training data. A forest of decision trees is randomly created, and the mean prediction of the individual trees is determined. There is a direct correlation between the number of trees in the forest and the accuracy of the results that are generated. The accuracy of the results is increased by increasing the number of trees. RF has several benefits such as being able to work with missing values and analysis of categorical values [5]. Support Vector Machine (SVM) is first fed with labelled data (supervised learning) permitting identification of the different groups and from this it builds a model for distinguishing the groups. Subsequently when provided with unlabelled fresh data SVM develops models or hyperplanes to separate one group from another. SVM is capable of performing both regression and classification tasks and can handle both continuous and categorical variables [6] SVM is resistant to overfitting, which is a risk in analysis of small datasets. Linear Discriminant Analysis (LDA) reduces the number of features or predictors need to accurately classify and discriminate the groups. This is desirable for our dataset as we start with close to 900,000 potential features to be used for CHD detection. LDA is simple in approach but it still achieves excellent accuracy. The accuracy achieved is similar to that obtained with more complex methods. LDA is based on the identification of a linear combination of variables (predictors) that best separates the two classes (targets) [7]. It is closely related to analysis of variance (ANOVA) and regression analysis which attempts to define an outcome variable based on a combination of explanatory variables. Partitioning Around Medoids (PAM) is a statistical technique for

class prediction from gene expression data using the nearest shrunken centroids [2, 8]. This method identifies the subsets of genes that best characterize each class. Generalized Linear Models (GLMs) are a broad class of models that include linear regression, ANOVA, Poisson regression, log-linear models and others [2, 8]. Deep Learning (DL) is a form of representation learning that uses multiple transformation steps to create very complex features. DL is categorized into feed-forward artificial neural networks (ANNs), which uses more than one hidden layer (y) that connects the input (x) and output layer (z) via a weight (W) matrix. The weight matrix is expected to minimize the difference between the input and output layers and is considered as the best AI approach [2, 8].

Modeling & Evaluation:

Two-step validation was utilized for these analyses. There were two different data sets, first was utilized to build the model and test it, the second one was used to validate the model.

While using the two-step validation method, two different techniques were utilized to find out the best model and calculate the performance metrics: 10-fold Cross validation and Bootstrapping.

1) **10-fold Cross Validation:** The first data set was split into a training to train the model first with a portion of the data and a test group (remaining portion) on which the performance of the developed model is then determined. Here we randomly divided the available set of samples into two parts: a training set and a test or hold-out set. The model was fit on the training set, and the fitted model was used to predict the responses for the observations in the hold-out set. Estimates were used to select best model, and to give an idea of the test error of the final chosen

model. Idea was to randomly divide the data into 10 equal-sized parts. We left out part 10, fit the model to the other 9 parts (combined), and then obtained predictions for the left-out 10th part. This was done in turn for each part k = 1, 2...10, and then the results were combined. This process was repeated a total of ten times and the average AUC, sensitivity, specificity and 95% confidence intervals for the test set were calculated. Then, as the validation step, AUC, sensitivity, specificity and 95% confidence intervals for the validation data set were calculated, too.

2) **Bootstrapping:** The bootstrap is a flexible and powerful statistical tool that allowed us to use a computer to mimic the process of obtaining new data sets, so that we were able to estimate the variability of our estimate without generating additional samples. Rather than repeatedly obtaining independent data sets from the population, we instead obtained distinct data sets by repeatedly sampling observations from the original data set with replacement. Each of these “bootstrap data sets” was created by sampling with replacement and was the same size as our original dataset. As a result, some observations appeared more than once in each bootstrap data set and some not at all. To estimate prediction error using the bootstrap, we used each bootstrap dataset as our training sample, and the original sample as our test sample. This process was repeated a total of ten times and the average AUC, sensitivity, specificity and 95% confidence intervals for the test set were calculated. Then, as the validation step, AUC, sensitivity, specificity and 95% confidence intervals for the validation data set were calculated, too.

References:

1. Bahado-Singh RO, Vishweswaraiah S, Aydas B, Mishra NK, Guda C, Radhakrishna U. Deep Learning/Artificial Intelligence and Blood-Based DNA Epigenomic Prediction of Cerebral Palsy. International Journal of Molecular Sciences 2019;20:2075.
2. Alakwaa FM, Chaudhary K, Garmire LX. Deep Learning Accurately Predicts Estrogen Receptor Status in Breast Cancer Metabolomics Data. J Proteome Res 2018;17:337-47.
3. Bahado-Singh RO, Vishweswaraiah S, Er A, et al. Artificial Intelligence and the detection of pediatric concussion using epigenomic analysis. Brain research 2020;1726:146510.
4. Bahado-Singh RO, Vishweswaraiah S, Aydas B, et al. Artificial intelligence and leukocyte epigenomics: Evaluation and prediction of late-onset Alzheimer's disease. 2021;16:e0248375.
5. Huang JH, Xie HL, Yan J, Lu HM, Xu QS, Liang YZ. Using random forest to classify T-cell epitopes based on amino acid properties and molecular features. Anal Chim Acta 2013;804:70-5.
6. Mahadevan S, Shah SL, Marrie TJ, Slupsky CM. Analysis of metabolomic data using support vector machines. Anal Chem 2008;80:7562-70.
7. Liland KH. Multivariate methods in metabolomics – from pre-processing to dimension reduction and statistical analysis. TrAC Trends in Analytical Chemistry 2011;30:827-41.
8. Candel A, Parmar V, LeDell E, Arora A. *Deep Learning with H2O.* Number of pages.
