# Supplementary figures and images for: Artificial Intelligence and Circulating Cell-Free DNA Methylation Profiling: Mechanism and Detection of Alzheimer’s Disease
Source: Cells. 2022 May 25;11(11):1744. doi: 10.3390/cells11111744 (PMC9179874; doi:10.3390/cells11111744)

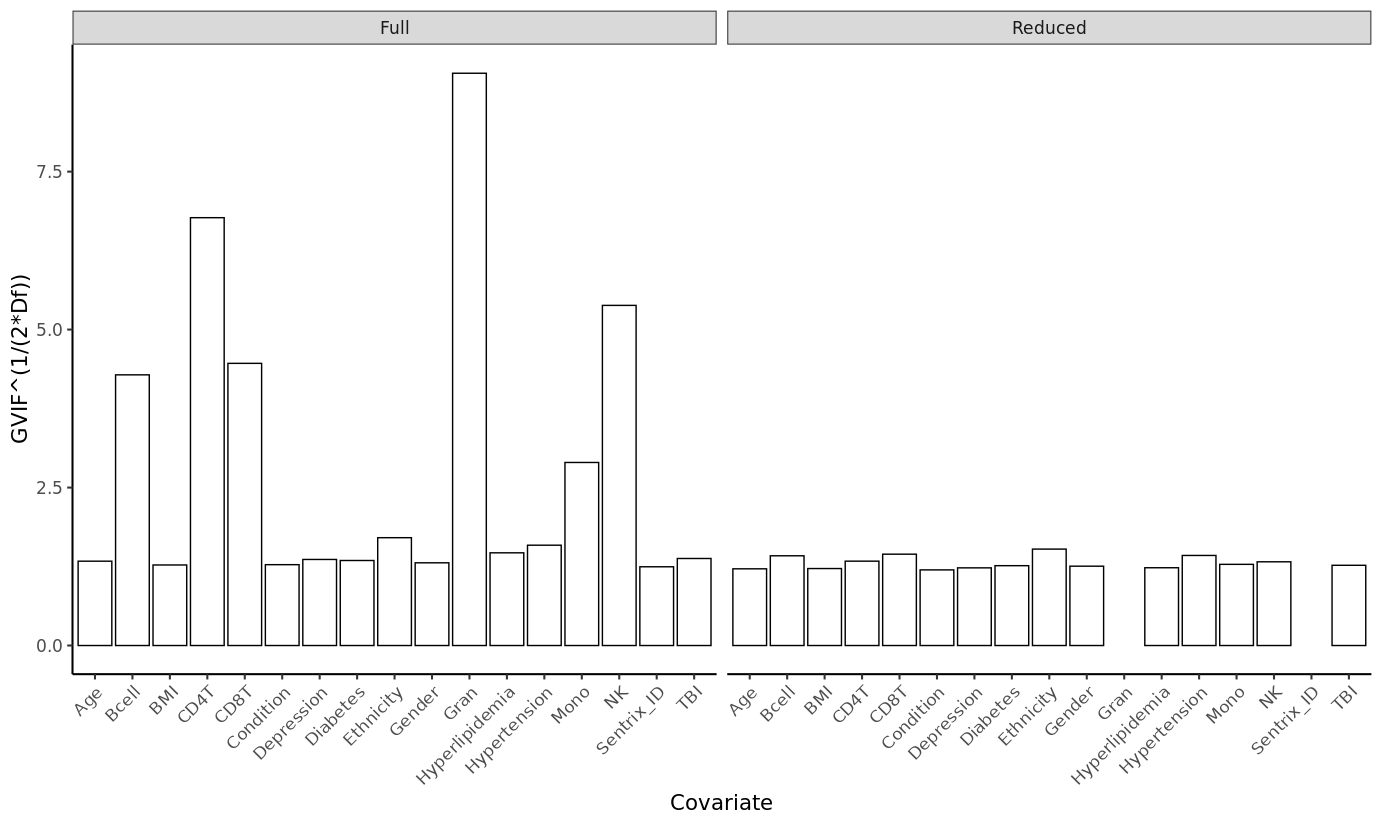

Supplement: Supplementary file 1 [file cells-11-01744-s001.zip › Supp Figure S1.png]

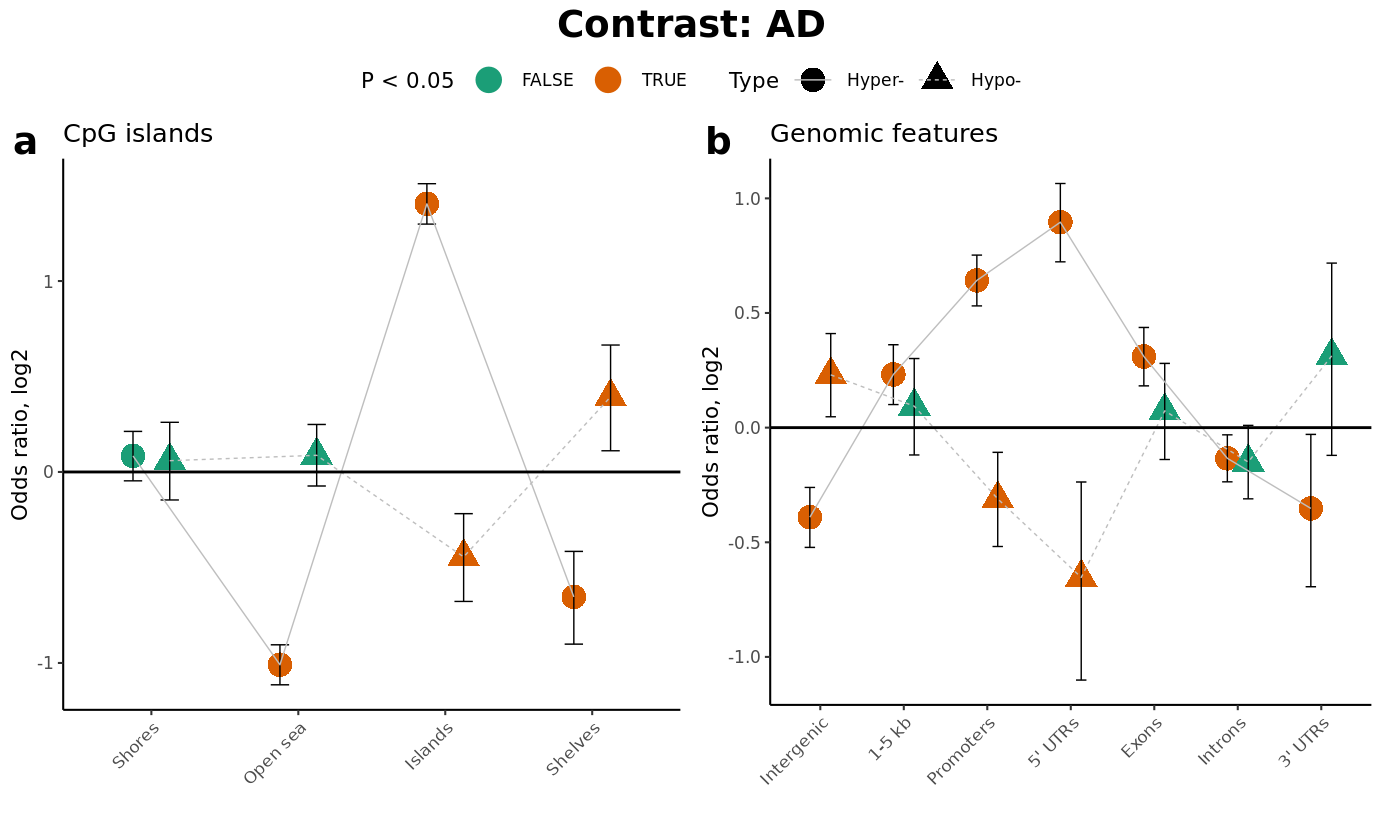

Supplement: Supplementary file 1 [file cells-11-01744-s001.zip › Supp Figure S2.png]

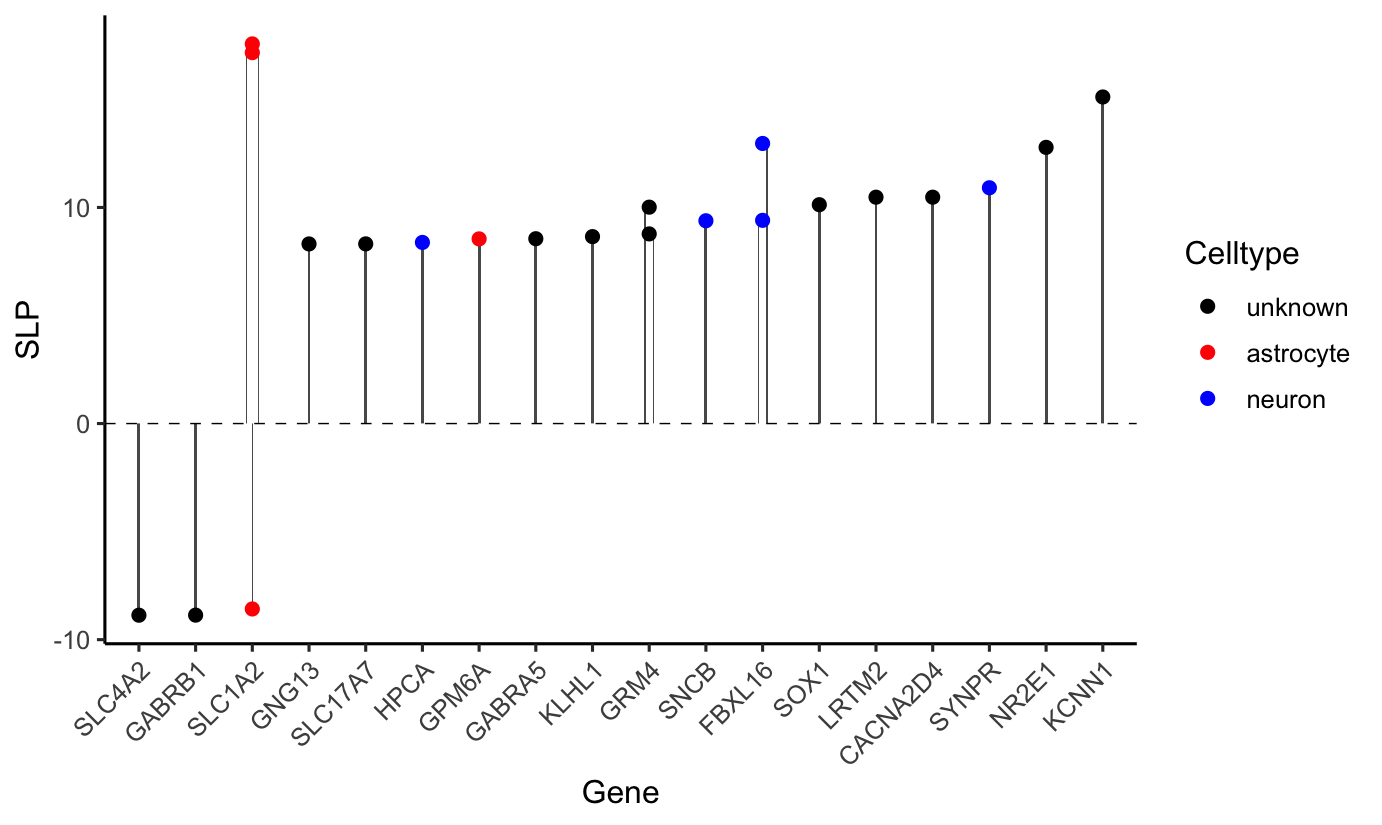

Supplement: Supplementary file 1 [file cells-11-01744-s001.zip › Supp Figure S3.png]
